# Optimal configuration method of demand-side flexible resources for enhancing renewable energy integration

**DOI:** 10.1038/s41598-024-58266-6

**Published:** 2024-04-01

**Authors:** Yu Fu, Hao Bai, Yongxiang Cai, Weichen Yang, Yue Li

**Affiliations:** 1 Electric Power Research Institute of Guizhou Power Grid Co., Ltd., Guizhou Power Grid Co., Ltd., Guiyang, China; 2 Institute of Power Distribution Technology, Electric Power Research Institute of South Power Grid Co., Ltd., Guangzhou, China

**Keywords:** Demand-side flexible resources, Generalized energy storage, Optimize configuration, Renewable energy consumption, Electrical and electronic engineering, Energy grids and networks

## Abstract

Demand-side flexible load resources, such as Electric Vehicles (EVs) and Air Conditioners (ACs), offer significant potential for enhancing flexibility in the power system, thereby promoting the full integration of renewable energy. To this end, this paper proposes an optimal allocation method for demand-side flexible resources to enhance renewable energy consumption. Firstly, the adjustable flexibility of these resources is modeled based on the generalized energy storage model. Secondly, we generate random scenarios for wind, solar, and load, considering variable correlations based on non-parametric probability predictions of random variables combined with Copula function sampling. Next, we establish the optimal allocation model for demand-side flexible resources, considering the simulated operation of these random scenarios. Finally, we optimize the demand-side resource transformation plan year by year based on the growth trend forecast results of renewable energy installed capacity in Jiangsu Province from 2025 to 2031.

## Introduction

In the context of an energy crisis and environmental pollution, China has proposed constructing a new power system with renewable energy as the main source. Wind power, photovoltaic, and other renewable energy sources are rapidly advancing. However, due to the increased integration of a high proportion of renewable energy, its volatility and uncertainty have led to a significant rise in the demand for power system flexibility. This poses substantial risks to the safe and stable operation of the system. Therefore, it is urgent to fully tap into the flexible adjustment potential of each aspect, including generation, transmission, consumption, and storage, establishing a balance between demand and supply of flexibility^1^. This is crucial to support the consumption of a high proportion of renewable energy and ensure the economic and safe operation of the system.

Flexibility aggregation, seen as a promising solution to invoke system flexibility resources, has been extensively applied in various cluster studies such as electric vehicles (EVs), temperature-controlled loads, and micro energy networks^2–5^. In reference^6^, a generalized battery model is formulated, and the aggregated flexibility of controllable temperature loads is accurately calculated using power and capacity bounds derived from the continuous model. References^7,8^ apply a geometric approach to represent individual cells as polyhedral feasible sets, and then aggregate the operating power of controllable temperature loads and distributed generation based on Minkowski sums. In reference^9^, a real-time aggregation method based on reinforcement learning is proposed for fast aggregation of electric vehicles' flexibility feedback design. Reference^10^ simplifies the constraints of the aggregated feasible domain using k-order approximation and multi-timescale approximation models, aiming to reduce the complexity of large-scale multi-energy flexibility load aggregation, thereby enhancing computational speed while maintaining result accuracy.

Based on the aggregation of flexibility resources, references^11–13^ have conducted studies on the participation of flexibility resources in grid optimization and dispatch. On the other hand, reference^14^ fits the conditional probability distribution of wind power prediction error through nonparametric kernel density estimation. It applies numerical methods to obtain the upper and lower bounds of wind power prediction error under a certain level of confidence, quantifying it as flexibility demand. Reference^15^, treats the forecast error of renewable energy and load-shedding due to system failures as flexibility demands. Reference^16^ develops a robust optimization model to address prediction errors in wind power by allocating reserves and energy storage (ES) for conventional units. Reference^17^ uses the fluctuating power of net load, obtained based on the power of wind turbines, PV, and loads on a typical day, as a flexibility demand. Additionally, reference^18^ incorporates the fluctuation of the net load forecast value in the neighboring moment and the net load output interval of the next moment as the flexibility demand. Reference^19^ deems this approach not comprehensive enough and expresses the flexibility demand as the fluctuation of the net load forecast value in the neighboring moment plus the net load output interval of the current and next moments. Moreover, reference^20^ derives the flexibility demand envelope based on the probability distribution of the fluctuating power of wind power at different time scales. However, none of these studies considered the conditional probability distribution of wind power fluctuation power. In contrast, references^21,22^ determine the variation interval of wind power fluctuation power as flexibility demand based on the conditional probability distribution of the actual fluctuation power of wind power. However, the failure to analyze relevant quantities of wind power fluctuation might decrease the accuracy of the conditional probability distribution model, leading to inaccuracies in the obtained wind power fluctuation power interval. In summary, the current stage of studies on the quantification of flexibility demand primarily focuses on either uncertainty or volatility individually, without comprehensive consideration of both. Moreover, there is limited research on the conditional probability of wind power wave momentum.

Compared with direct control for demand-side flexible resources, indirect control method based on dynamic pricing reserves more autonomy for end users. The utility company or aggregator sets the electricity prices, and the consumers respond to the prices by adjusting the amount of energy they use. Various retail pricing models have been investigated in the existing literature. The dynamic pricing mechanisms, which denote the time varying pricing schemes^23–26^, are well adopted due to the high efficiency. In^23^, the authors investigate the required information and communications systems that are needed to realize the control-by-price concept for such units. The dynamic pricing algorithm is also used in demand response programs to maximize the retailer’s profit^24–26^.

In the context of a high proportion of wind power integration, the demand for flexibility is growing. Consequently, it is necessary to conduct reasonable capacity planning for flexibility resources and ensure adequate resource configuration to maximize their regulatory role through optimal scheduling. This will effectively enhance system flexibility, reduce the likelihood of wind and solar energy curtailment, and promote the utilization of renewable energy.

The unit's output and rotational reserve are the most conventional flexibility resources. Currently, research on unit scheduling has delved deeper. Reference^27–29^ proposed a unit combination method for grid-connected wind power, considering the prediction error of wind power generation and the uncertainty of system operation in the optimization model. Reference^30^ established a robust unit combination model incorporating transmission constraints and introduced a cost evaluation method to mitigate model conservatism. Reference^31^ adopted the objective of maximizing social benefit to achieve optimal unit combination, considering demand-side response. Additionally, reference^32^ integrated the operation risk model with the unit combination model for synergistic optimization of operation cost and risk.

In the context of optimal scheduling with storage, some studies focus on the joint operation optimization of storage and other resources. Reference^33^ optimized the scheduling of a coupled PV-storage system under various scenarios. Reference^34^ comprehensively considered multiple resources, including distributed generation, energy storage, and controllable load, utilizing two models for flexibility regulation. Capacity-rich resources were directly involved, while capacity-scarce resources responded based on tariff incentives. A joint optimal scheduling model was established by combining these two models. Reference^35^ proposed a two-phase optimization model for energy storage to maximize the integrator's profit while considering the uncertainty of customer demand.

Several studies have applied energy storage in a narrowly defined flexibility demand scenario, particularly in peaking scenarios. References^36,37^ investigated the economic potential of utilizing energy storage to provide peaking capacity in shorter time scales. Reference^38^ argued that configuring energy storage on the thermal power plant side is akin to increasing the depth of thermal power unit peaking. They established an optimized scheduling model for energy storage, thermal power units, and demand-side response, comprehensively considering the deep peaking initiative of thermal power units configured with energy storage and the moderating role of demand-side response. However, optimizing the dispatch of thermal power plants and energy storage to maximize power sent to the electricity market for higher revenue, ensuring sufficient reserve capacity, and fully developing the reserve potential of energy storage to optimize unit capacity allocation remains a pressing issue to be addressed.

In addition to the two types of conventional flexibility resources, namely unit output and energy storage, some literature has also explored the optimal scheduling of other flexibility resources such as interruptible loads and electric vehicles. Reference^39^ proposes three flexibility evaluation indices that can characterize the flexibility of distribution networks and establishes a two-stage flexibility enhancement optimization model for distribution networks, integrating EV charging with energy storage and interruptible load scheduling. Reference^40^ considers heat pumps as a flexibility resource and establishes a day-ahead optimal dispatch model based on cooperative game theory for distribution networks. Reference^41^ uses a virtual battery model to represent uncertain clusters of EVs and considers their flexibility, optimizing the day-ahead power generation and standby capacity of EVs to assist in regulating the operational flexibility of the system. Reference^42^ explores the flexibility of soft switches in the distribution network and establishes an optimal scheduling model including soft switches, taking into account the operational constraints of soft switches and aiming to minimize operational costs while improving the system's flexibility. An optimal scheduling model including soft switches is established.

The studies mentioned above have focused on aggregating flexibility resources and optimizing dispatching. However, few studies have addressed the optimal configuration of flexibility resources.

In this paper, a demand-side flexible resource optimal allocation method for renewable energy consumption enhancement is proposed. The main contributions of this work are three-fold.

- To reduce the complexity of optimization model when dealing with numerous demand-side flexible devices, generalized energy storage model is adopted to characterize the aggregate flexibility of demand-side resource cluster;
- To consider the correlations among different random factors such as wind, photovoltaic and load, when generating stochastic scenarios for robust optimization, a non-parametric probability prediction method based on Copula function is developed;
- For obtaining an optimal demand-side flexible resources configuration scheme with high robustness against uncertainties, a robust optimization method based on stochastic scenario traversal is proposed. Besides, the effectiveness of the proposed method is evaluated based on a practical case in Jiangsu, China.

The rest of the paper is organized as follows. Section "Aggregate flexibility modeling for demand-side resources" presents the aggregate flexibility modeling method for demand-side resources. Section "Scenario generation considering wind, solar, and load correlations" introduces the scenario generation method considering wind, solar, and load correlations. The proposed optimal configuration method of demand-side flexible resources are explained in detail in Section "Robust optimal configuration method of demand-side flexible resources". In Section "Example analysis", the effectiveness of the proposed method is validated on real-world cases. Finally, Section "Conclusion" concludes the paper.

## Aggregate flexibility modeling for demand-side resources

The proportion of Flexible Load (FL) with regulation capability on the demand side is steadily increasing. Loads that can alter electricity consumption patterns are referred to as demand-side resources (DSRs). Numerous scholars and experts worldwide have conducted relevant research on assessing the potential response of demand-side resources. When the grid experiences failures or encounters power supply inadequacies during peak periods, demand response utilizing controllable resources can achieve short-term load reduction. This helps maintain a balance between supply and demand on the grid and enhances the grid's operational quality. Temperature-controlled loads and electric vehicles possess significant response capacities, rapid response speeds, and good adjustability, making them valuable fast-response resources.

For electric vehicles and air conditioners, categorized as demand-side flexible resources with time-coupled characteristics, they demonstrate charging, discharging, and storage traits akin to energy storage. Instead of fixed boundary parameters as seen in traditional energy storage models, we employ time-varying power and energy boundaries. This accounts for the fact that loads like electric vehicles and air conditioners need to adhere to user comfort constraints during operation. Building upon the generalized energy storage model that encompasses individual devices, we derive the aggregated flexibility model of demand-side flexible resources by calculating the geometric centers of all device parameters.

For facilitating the management of these flexible DSRs, DSR aggregators are introduced to aggregates these massive DSRs and represent them in interactions with the electricity market and power system operators. In this paper, the DSR owners (i.e., users) enter into long-term contracts with aggregators. The contracts stipulate that users can access electricity at a rate lower than the general level, while the aggregators, through direct control means, conduct energy arbitrage by utilizing flexible resources, ensuring user comfort, travel needs, and other constraints are met.

### Generalized battery model

The generalized battery model (GBM) includes the energy storage state change equation, energy constraint and power constraint:

1a $$e_{i,t} = \rho_{i} e_{i,t - 1} + \Delta e_{i,t} + \left\{ {\begin{array}{*{20}l} {\Delta t \cdot \eta_{i}^{{\text{ in }}} \cdot p_{i,t} ,p_{i,t} > 0} \hfill \\ {\Delta t \cdot p_{i,t} /\eta_{i}^{{\text{ out }}} ,p_{i,t} < 0} \hfill \\ \end{array} } \right.$$

1b $$e_{i,t}^{V} \le e_{i,t} \le e_{i,t}^{ \wedge }$$

1c $$p_{i,t}^{V} \le p_{i,t} \le p_{i,t}^{ \wedge }$$

where: $e_{i,t}$ denotes the energy of device $i$ at moment $t$; $\rho_{i}$ denotes the energy decay coefficient of device $i$; $\Delta e_{i,t}$ denotes the energy change of device $i$ at moment $t$; $p_{i,t}$ denotes the power of $i$ at moment $t$; $e_{i,t}^{ \wedge }$ and $e_{i,t}^{V}$ denote the energy boundary of device $i$ at moment $t$; $p_{i,t}^{ \wedge }$ and $p_{i,t}^{V}$ denote the power boundary of device $i$ at moment $t$; $\eta_{i}^{{\text{ in }}}$ in and $\eta_{i}^{{\text{ out }}}$ denote the charging and discharging efficiency of device $i$ at moment $t$, respectively.

### Electric vehicle

The large-scale adoption of electric vehicles is of paramount importance in reducing fossil fuel consumption and safeguarding the environment. The widespread proliferation of electric vehicles will result in a significant increase in electricity demand. Electric vehicles, known for their energy efficiency and environmental friendliness, can serve as unconventional energy storage devices, actively participating in demand response and offering auxiliary services to the power grid. Strategically planning the charging and discharging cycles of these vehicles can help smooth out peak demand, minimize troughs, stabilize the system, and effectively ease the burden on the power grid. Investigating the charging patterns of electric vehicles enables a comprehensive understanding of their charging habits. With the advancement of Vehicle-to-Grid (V2G) technology, electric vehicles can interact with microgrids to exchange energy. Leveraging the energy storage capabilities of electric vehicles can not only relieve stress on the power grid but also provide advantages to vehicle owners. Thus, to optimize the utilization of electric vehicle energy storage capabilities, accurate prediction of charging loads and an in-depth study of charging behavior are imperative.

Before calculating the GBM parameter set $\{{p}_{t}^{\bigwedge },{p}_{t}^{\bigvee },{e}_{t}^{\bigwedge },{e}_{t}^{\bigvee },\Delta {e}_{t}\}$ for each individual EV, it is essential to introduce a fundamental knowledge: for vehicle owners, the most preferred charging trajectory is when the electric vehicle charges at the maximum power until the battery is fully charged. In this paper, we refer to it as the optimal charging trajectory. On the other hand, the least acceptable charging trajectory for vehicle owners is when the electric vehicle charges at the slowest speed until departing, ensuring the battery is fully charged before leaving. In this paper, we refer to it as the worst charging trajectory. Any charging trajectories within the region bounded by the optimal charging trajectory and the worst charging trajectory is acceptable for users. Therefore, the energy state corresponds to the best/worst charging trajectory is regarded as the upper/lower boundary of the GBM, which is calculated by Eq. (2). The power boundary is jointly determined by the GBM energy boundaries and the battery rated power, which is calculated by Eq. (3). The energy change of the GBM is defined as the remaining battery energy when plugged into the charging pile or the battery energy when leaving the charging pile, which is calculated by Eq. (4).

#### Energy boundaries

For each electric vehicle, in order to meet the charging needs of users, the upper and lower energy limits at each moment can be calculated using Eq. (2):

2a $$e_{i,t}^{ev, \wedge } = \min \left\{ {\left( {e_{{i,ta_{i} }}^{ev} + \overline{p}_{i}^{ev} (t - ta_{i} )\Delta t} \right),\overline{e}_{i}^{ev} } \right\},t \in \left( {ta_{i} , \ldots ,tl_{i} } \right)$$

2b $$e_{i,t}^{ev, \min 1} = \max \left\{ {\left( {e_{i}^{ev,\exp } - \overline{p}_{i}^{ev} (tl_{i} - t)\Delta t} \right),\underline {e}_{i}^{ev} } \right\},\,t \in \left( {ta_{i} , \ldots ,tl_{i} } \right)$$

2c $$e_{i,t}^{ev, \min 2} = \max \left\{ {\left( {e_{{i,ta_{i} }}^{ev} - \overline{p}_{i}^{ev} (t - ta_{i} )\Delta t} \right),\underline {e}_{i}^{ev} } \right\},\,t \in (ta_{i} , \ldots ,tl_{i} )$$

2d $$e_{i,t}^{ev, \vee } = \max \left( {e_{i,t}^{ev, min 1} ,e_{i,t}^{ev, min 2} } \right),\,t \in \left( {ta_{i} , \ldots ,tl_{i} } \right)$$

where Eq. (2a) represents the upper limit of the electric vehicle's power at each moment; Eq. (2b) represents the power curve corresponding to the time when the electric vehicle starts charging from the lowest power and charges to the target power when it leaves the station, Eq. (2c) represents the power curve corresponding to the time when the electric vehicle arrives at the station and starts discharging until it reaches the lowest power, and Eq. (2d) represents the electric vehicle's lower limit of the electric vehicle's power at each moment, and it can be calculated to The lower limit of the electric vehicle's power at each moment is obtained. Where: $e_{i,t}^{ev, \wedge / \vee }$ denote the upper/lower energy boundaries of EV; $ta_{i}$, $tl_{i}$ denotes the driving-in/driving-out time of EV; $\overline{e}_{i}^{ev}$/$\underline {e}_{i}^{ev}$ denotes the upper and lower limits of battery capacity of EV; and $\overline{p}_{i}^{ev}$ denotes the rated power of EV.

#### Power boundaries

For each electric vehicle, the upper and lower limits of charging and discharging power are limited by both the energy limit and the power rating:

3a $$p_{i,t}^{ev, \wedge } = \min \left\{ {\left( {e_{i,t + 1}^{ev, \wedge } - e_{i,t}^{ev, \vee } } \right)/\Delta t,\overline{p}_{i}^{ev} } \right\}\,t \in (ta_{i} , \ldots ,tl_{i} )$$

3b $$p_{i,t}^{ev, \vee } = \min \left\{ {\left( {e_{i,t + 1}^{ev, \vee } - e_{i,t}^{ev, \wedge } } \right)/\Delta t, - \overline{p}_{i}^{ev} } \right\}\,t \in (ta_{i} , \ldots ,tl_{i} )$$

where Eq. (3a) denotes the upper power limit of EV and Eq. (3b) denotes the lower power limit of EV. Where: $p_{i,t}^{ev, \wedge / \vee }$ denote the upper/lower power boundaries of the electric vehicle; $ta_{i}$, $tl_{i}$ denotes the electric vehicle drive-in/drive-out time; and $\overline{p}_{i}^{ev}$ denotes the rated power of the electric vehicle.

#### Energy change

The energy change due to EVs leaving or entering the station can be calculated based on the state of the EV at the station, the initial energy at the time of entering the station, and the final energy at the time of leaving the station:

4 $$\Delta e_{t + 1}^{ev} = e_{i,t + 1}^{ev, \max } \cdot (x_{i,t + 1} - x_{i,t} ) - e_{i,t}^{ev, \max } \cdot (x_{i,t} - x_{i,t + 1} )$$

where: $\Delta e_{t}^{ev }$ denotes the amount of transferred electricity due to EVs leaving or entering the station; $x_{i,t}$ denotes the state of EVs at the station.

### Air conditioner

Air conditioners, being the most prevalent flexible loads, have the ability to convert electrical energy into heat or refrigeration for short-term storage. The output of air conditioning loads is influenced by seasonal and weather conditions, resulting in significant loads during winter and summer, and lighter loads during spring and fall. Loads are higher during extreme temperatures and lower during moderate ones, closely related to human body temperature. The energy storage features of air conditioning can be effectively utilized for regulation and control, thus serving the microgrid as an energy storage device.

To determine the GBM parameter set $\{{p}_{t}^{\bigwedge },{p}_{t}^{\bigvee },{e}_{t}^{\bigwedge },{e}_{t}^{\bigvee }\}$ for each individual AC, several optimization problems are formulated. In detail, the power boundaries, ${p}_{t}^{\bigwedge }$ and ${p}_{t}^{\bigvee }$, correspond to the minimum and maximum power consumption of the AC while ensuring the user's thermal comfort constraints, which is calculated by Eqs. (5) and (6). Additionally, the energy boundaries, ${e}_{t}^{\bigwedge }$ and ${e}_{t}^{\bigvee }$, represent the energy levels at which the indoor temperature is maintained at the maximum acceptable and minimum acceptable levels, respectively. They are calculated by solving the optimization problem (7).

#### Reference power

To obtain the baseline power of a single air conditioner, an optimization model is established with the objective of minimizing the temperature deviation, considering the user comfort constraints and the dynamic equations of building room temperature:

5a $$p_{i,t}^{{\text{ hvac,base }}} = \mathop { \arg\min }\limits_{{\{ P_{i,t}^{{\text{ hvac }}} \}_{t \in T} }} \sum\limits_{t \in T} {\left( {\theta_{i,t} - \theta_{i}^{{\text{ set }}} } \right)^{2} }$$

5b $$\theta_{i,t} = a \cdot \theta_{i,t - 1} + (1 - a) \cdot \left( {\theta_{i,t - 1}^{ \, out \, } - b \cdot P_{i,t - 1}^{hvac} } \right)$$

5c $$\theta_{i,t}^{{\text{ min }}} \le \theta_{i,t} \le \theta_{i,t}^{ max } \forall t \in T$$

5d $$\theta_{i,t}^{{{\text{ min }}/{\text{ max }}}} = \theta_{i}^{{\text{ set }}} \pm \Delta \theta_{i} \forall t \in T$$

5e $$0 \le p_{i,t}^{{\text{ hvac }}} \le \overline{p}_{i,t}^{{\text{ hvac }}} \forall t \in T$$

where (5a) denotes the optimization objective, i.e., the indoor temperature deviation from the user's temperature setpoint; (5b)–(5e) denote the constraints of the optimization model, where (5b) denotes the discrete form of the temperature variation equation, (5c) denotes the indoor temperature constraints, (5d) denotes the maximum and minimum temperature acceptable to the user and (5e) denotes the power constraints. Where: $T = \left\{ {1, \cdots 24} \right\}$ denotes a discrete set of time points; $P_{i,t}^{{\text{ hvac,base }}}$ denotes the base power of the air conditioner to maintain the indoor temperature at the user set temperature; $\overline{P}_{i,t}^{{\text{ hvac }}}$ denotes the rated power of the air conditioner; $\theta_{i}^{{\text{ set }}}$, $\theta_{i,t}$, $\theta_{i,t}^{{\text{ out }}}$, $\Delta \theta_{i}$ denote the user set temperature, the indoor temperature, the outdoor temperature, and the maximum deviation, respectively; and a, b denote the coefficients of the equation of variation of the indoor temperature, which are related to the equivalent heat capacity (C), the equivalent thermal resistance (R), and the operating efficiency (η) as:

5f $$a = e^{ - \Delta t/(RC)} ,\,b = R\eta$$

#### Power boundaries

In order to obtain the maximum/minimum power limits for a single air conditioner, an optimization model is built with the objective of minimum or maximum power consumption at each moment, respectively:

6a $$\begin{gathered} p_{i,t}^{{\text{ hvac,min }}} = \mathop { \arg \min }\limits_{{\{ P_{i,t}^{{\text{ hvac }}} \}_{t \in T} }} p_{i,t}^{{\text{ hvac }}} \hfill \\ {\text{or }}p_{i,t}^{{\text{ hvac,max }}} = \mathop { \arg \max }\limits_{{\{ P_{i,t}^{{\text{ hvac }}} \}_{t \in T} }} p_{i,t}^{{\text{ hvac }}} \hfill \\ {\text{s}}.{\text{t}}.: \quad \quad \left( {{\text{5b}}} \right) - \left( {{\text{5e}}} \right) \hfill \\ \end{gathered}$$

After obtaining the minimum or maximum value of the power consumption at each moment, the adjustable power upper and lower bounds of the aggregation model can be obtained by subtracting the baseline power:

6b $$p_{i,t}^{{{\text{ hvac,}} \vee \, }} = p_{i,t}^{{\text{ hvac,min }}} - p_{i,t}^{{\text{ hvac,base }}}$$

6c $$p_{i,t}^{{{\text{ hvac,}} \wedge \, }} = p_{i,t}^{{\text{ hvac,max }}} - p_{i,t}^{{\text{ hvac,base }}}$$

where: $P_{i,t}^{{{\text{ hvac,}} \vee {/} \wedge }}$ denotes the lower and upper adjustable power limits of the air conditioner.

#### Energy boundaries

When the aggregation model state is at the upper and lower energy boundaries, the indoor temperature is considered to be at the boundary of the user's acceptable range. Therefore, when calculating the energy boundaries of the aggregation model, the optimization model is built with the objective of minimizing the deviation value between the indoor temperature and the maximum/minimum acceptable temperature, respectively:

7a $$\begin{gathered} p_{i,t}^{{\text{ hvac, - }}} = \mathop { \arg\min }\limits_{{\{ P_{i,t}^{{\text{ hvac }}} \}_{t \in T} }} (\theta_{i,t} - \theta_{i,t}^{{\text{ max }}} )^{2} \hfill \\ {\text{or }}p_{i,t}^{{\text{ hvac, + }}} = \mathop { \arg\min }\limits_{{\{ P_{i,t}^{{\text{ hvac }}} \}_{t \in T} }} (\theta_{i,t} - \theta_{i,t}^{{\text{ min }}} )^{2} \hfill \\ {\text{s}}.{\text{t}}.: \quad \quad \left( {{\text{5b}}} \right) - \left( {{\text{5e}}} \right) \hfill \\ \end{gathered}$$

After obtaining the power values $P_{i,t}^{{\text{ hvac, - }}}$ and $P_{i,t}^{{\text{ hvac, + }}}$ corresponding to the maximum/minimum temperatures, the upper and lower bounds of the energy of the aggregation model can be obtained by substituting the following equations:

7b $$e_{i,t}^{{{\text{ hvac,}} \vee \, }} = \frac{{\sum\nolimits_{t = 0}^{T - 1} {\left( {p_{i,t}^{{\text{ hvac, - }}} - p_{i,t}^{{{\text{ hvac }},{\text{ base }}}} } \right) \cdot \Delta t} }}{{\left( {1 - a} \right)T}}\forall t \in T$$

7c $$e_{i,t}^{{{\text{ hvac,}} \wedge \, }} = \frac{{\sum\nolimits_{t = 0}^{T - 1} {\left( {p_{i,t}^{{\text{ hvac, + }}} - p_{i,t}^{{{\text{ hvac }},{\text{ base }}}} } \right) \cdot \Delta t} }}{{\left( {1 - a} \right)T}}\forall t$$

where: $e_{i,t}^{{{\text{ hvac,}} \vee {/} \wedge }}$ indicates the min/max power of the air conditioner.

### Aggregate flexibility modeling

When performing aggregation flexibility modeling, we obtain the parameters of the aggregates by directly summing or computing a weighted average of the parameters of the generalized energy storage model corresponding to the individual devices.

8a $$\rho_{agg} = \sum\limits_{{i \in \Phi_{agg} }} {(\omega_{i} \rho_{i} )}$$

8b $$\eta_{agg}^{{\text{ in/out }}} = \sum\limits_{{i \in \Phi_{agg} }} {(\omega_{i} \eta_{i}^{{\text{ in/out }}} )}$$

8c $$E_{agg,t}^{v/ \wedge } = \sum\limits_{{i \in \Phi_{agg} }} {(e_{i,t}^{v/ \wedge } )}$$

8d $$P_{agg,t}^{v/ \wedge } = \sum\limits_{{i \in \Phi_{agg} }} {(p_{i,t}^{v/ \wedge } )}$$

8e $$\Delta E_{agg,t} = \sum\limits_{{i \in \Phi_{agg} }} {(\Delta e_{i,t} )}$$

where: $\Phi_{agg}$ denotes the set of all devices contained in the aggregator; $\omega_{i}$ denotes the weighting factor of device i within the aggregator.

## Scenario generation considering wind, solar, and load correlations

Wind power and photovoltaic power depend directly on natural meteorological conditions, resulting in natural uncertainty. Additionally, load is influenced by meteorological conditions, production, and lifestyle factors, further contributing to uncertainty. The combination of these factors amplifies the uncertainty in the distribution network trends, particularly in geographically similar regions. Within the same wind zone, wind turbines and photovoltaic equipment exhibit a correlation in power output. Similarly, in the same radiation zone, there is a strong correlation between wind speed, solar intensity, and load. These uncertainties and correlations significantly impact the operation of the distribution network. Therefore, it is crucial to consider randomized scenario sampling that accounts for the correlations among wind, solar, and load for both wind and solar power.

### Generation and load uncertainty modeling based on nonparametric probabilistic prediction

The existing forecasting methods for renewable energy and load are mainly categorized into point forecasting and probabilistic forecasting. Point prediction provides the single-point expected value of the forecasted object at a specific time in the future, yet it inherently incurs a prediction error due to its deterministic nature. On the other hand, probabilistic forecasting allows for the determination of the probability distribution associated with the forecasted object, enabling effective quantification of power system uncertainty. Within probabilistic prediction methods, nonparametric probabilistic prediction does not rely on assumptions about parametric probability distributions. This characteristic significantly enhances the accuracy of probabilistic prediction and forms a foundation for optimal decision-making in grid operations, taking uncertainty into account.

First, the historical data are normalized, and the direct quantile regression method is used to obtain the sequence of predicted quantile regression values, i.e., the discrete approximation form $\hat{F}_{t}$ of the cumulative probability distribution function, which can be expressed as:

9 $$\hat{F}_{t} = \left\{ {\hat{q}_{{\alpha_{k} ,t}} |0 \le \alpha_{1} < \cdots < \alpha_{k} < \cdots < \alpha_{m} \le 1} \right\}$$

where: $\hat{q}_{{\alpha_{k} ,t}}$, is the regression value of quantile ${q}_{{\alpha }_{k},t}$ at time $t$; $\alpha_{k}$ is the kth quantile; $m$ is the number of quantile points. The adjacent quantile points are approximated as random variables obeying a uniform distribution, and then an approximately complete probability distribution function $F_{{\text{t}}} \left( {x_{t} } \right)$ at each moment is obtained by the cubic interpolation method, where $x_{t}$ is the power of the predicted object at time $t$.

### Typical scenario generation considering source and load correlation

At the current stage, there are numerous reports on methods for generating random scenarios for a single variable, and these methods are relatively easy to implement. However, constructing and simulating joint distribution functions for multiple variables is challenging. The construction theory for most joint distribution functions is a simple extension of univariate distribution functions, often requiring all marginal distributions to follow the same distribution. In practical scenarios, it's difficult to satisfy such a strict requirement because different types of random factors, such as wind, solar, and load, typically follow different probability distributions. Addressing this issue, this paper uses Copula functions (link functions) to describe the correlation between wind, solar, and load, proposing a method based on Copula functions to generate random scenarios for wind, solar, and load. This method imposes no restrictions on the marginal distributions and can capture nonlinearity, asymmetry, and tail correlation relationships between variables.

Based on the probabilistic prediction results, we generate stochastic scenarios of renewable energy output, which can significantly reduce the difficulty of solving the problem by transforming the complex problem containing random variables into a deterministic optimization problem under each scenario when formulating the scheduling plan. Based on the Sklar theorem of multivariate distribution, the Copula function $C\left( \cdot \right)$ is used to construct the multivariate probability distribution function $F(x_{1} ,x_{2} , \ldots ,x_{T} )$ that takes into account the time dependence of PV output due to the correlation of renewable energy output, i.e.

10 $$F\left( {x_{1} ,x_{2} , \ldots ,x_{T} } \right) = C\left( {F_{1} (x_{1} ),F_{2} (x_{2} ), \ldots ,F_{T} (x_{T} )} \right)$$

where: $T$ is the duration of a scheduling cycle. By performing N times Monte Carlo sampling on the multivariate Copula function, we can obtain N renewable energy output scenarios with correlation. To avoid the huge renewable energy computational burden caused by the excessive number of scenarios, this paper clusters the generated scenarios to achieve scenario size reduction and obtain several typical PV output scenarios. In this paper, the K-Medoids clustering algorithm is used to cluster the scenes, which can avoid the clustering bias caused by the presence of anomalies, where the optimal number of clusters is determined by the combination of the contour coefficient method and the elbow method.

## Robust optimal configuration method of demand-side flexible resources

Since wind power, photovoltaic, and other renewable energy sources are significantly influenced by weather and environmental factors, the complexity of system planning increases upon their integration with the grid. Many scholars, both domestically and internationally, have extensively researched the uncertainty issue surrounding renewable energy. Ultimately, the solutions obtained from these efforts can be categorized into three groups: stochastic optimization, robust optimization, and fuzzy optimization. The stochastic optimization method possesses inherent limitations. Firstly, the probability density function, fundamental to stochastic optimization, is derived from fitting a vast amount of historical data. Secondly, as historical and sample data accumulates, the complexity of analysis escalates, and biased data can introduce errors in the analysis results. The fuzzy optimization method employs fuzzy numbers to represent uncertain variables and address the uncertainty issue. However, determining the affiliation function of fuzzy variables is challenging, resulting in a highly subjective and arbitrary affiliation function. The proposed robust optimization method overcomes the subjective and arbitrary nature of fuzzy optimization methods by defining the problem through the uncertainty set. It identifies the optimal solution that satisfies all specified conditions based on proposed constraints. In contrast to the previous two optimization methods, the robust optimization method does not necessitate fitting the distribution function of uncertain parameters using extensive historical data or constructing the affiliation function. It simply delineates the range of variation for each parameter to determine the optimal solution for the problem. Moreover, most decision schemes obtained through this method are robust against a range of disturbances.

In summary, the robust optimization method not only rectifies the shortcomings of the aforementioned two methods but also exhibits advantages that the other two methods lack. Simultaneously, the proposed robust optimization method contributes to enhanced operational efficiency.

### Optimization model

In the demand-side flexible resource optimal allocation model, the demand-side resource flexibility is modeled using a generalized energy storage model with the objective of minimizing the investment cost, while considering the power balance constraint of the system and the thermal generating unit output constraint. In addition to this, the constraints are simulated using N wind, solar and load day typical scenarios for the annual operation of the grid, with the aim of ensuring the robustness of the proposed configuration scheme, i.e., to be able to guarantee 100% grid consumption of renewable energy throughout the year. The specific optimization model is as follows:

11a $$min \left( {N_{HVAC} \cdot c_{HVAC} + N_{EV} \cdot c_{EV} + N_{ESS} \cdot c_{ESS} } \right)$$

11b $${\text{s}}.{\text{t}}.:\quad \begin{array}{*{20}c} {P_{s,t}^{RES} + P_{s,t}^{GEN} = P_{s,t}^{HVAC} + P_{s,t}^{HVAC,base} + P_{s,t}^{EV} + \cdot P_{s,t}^{ESS} P_{s,t}^{Load} } \\ {\forall s \in \Omega ,\forall t \in T} \\ \end{array}$$

11c $$\begin{gathered} E_{s,t}^{agg} = \rho_{s,t}^{agg} E_{s,t - 1}^{agg} + \Delta E_{s,t}^{agg} + \Delta t \cdot P_{s,t}^{agg} \hfill \\ \forall s \in \Omega ,\forall t \in T,\forall agg \in \{ HVAC,EV,\} \hfill \\ \end{gathered}$$

11d $$\begin{gathered} N_{agg} \cdot E_{t}^{agg,V} \le E_{s,t}^{agg} \le N_{agg} \cdot E_{t}^{agg,\Lambda } \hfill \\ \forall s \in \Omega ,\forall t \in T,\forall agg \in \{ HVAC,EV\} \hfill \\ \end{gathered}$$

11e $$N_{agg} \cdot P_{t}^{agg,v} \le P_{s,t}^{agg} \le N_{agg} \cdot P_{t}^{agg, \wedge }$$

11f $$\forall s \in \Omega ,\forall t \in T,\forall agg \in \{ HVAC,EV\}$$

11g $$P_{t}^{GEN,v} \le P_{s,t}^{GEN} \le P_{t}^{GEN,v}$$

where: $N_{HVAC}$, $N_{EV}$ denote the number of air conditioners and electric vehicles involved in the retrofit, respectively, and $N_{ESS}$ denote the quantity of renewable energy storage; $c_{HVAC}$, $c_{EV}$ denote the unit investment cost of air conditioners and electric vehicles involved in the retrofit, respectively, and $c_{ESS}$ denote the unit investment cost of energy storage; $P_{s,t}^{RES}$ denotes the power generation of renewable energy at moment $t$ under scenario $s$; $P_{s,t}^{GEN}$ denotes the generation capacity of thermal power plant at moment $t$ under scenario $s$. $P_{t}^{agg,v}$ and $P_{t}^{agg, \wedge }$ denote the minimum and maximum generation capacity constraints, respectively; $P_{s,t}^{agg}$ Represents the electricity consumption of flexible resources on the demand side at moment $t$ in scenario $s$. In the above optimization model, the objective function contains the retrofitting cost of installing intelligent control terminals for air conditioners and electric vehicles and the investment cost of adding renewable energy storage, and the constraints (11b) denote the power balance constraint; (11c)–(11e) denote the operation constraint of generalized energy storage, and (11g) denotes the generation capacity constraint of thermal power plants.

### Overall flow of the proposed method

**Figure 1:**
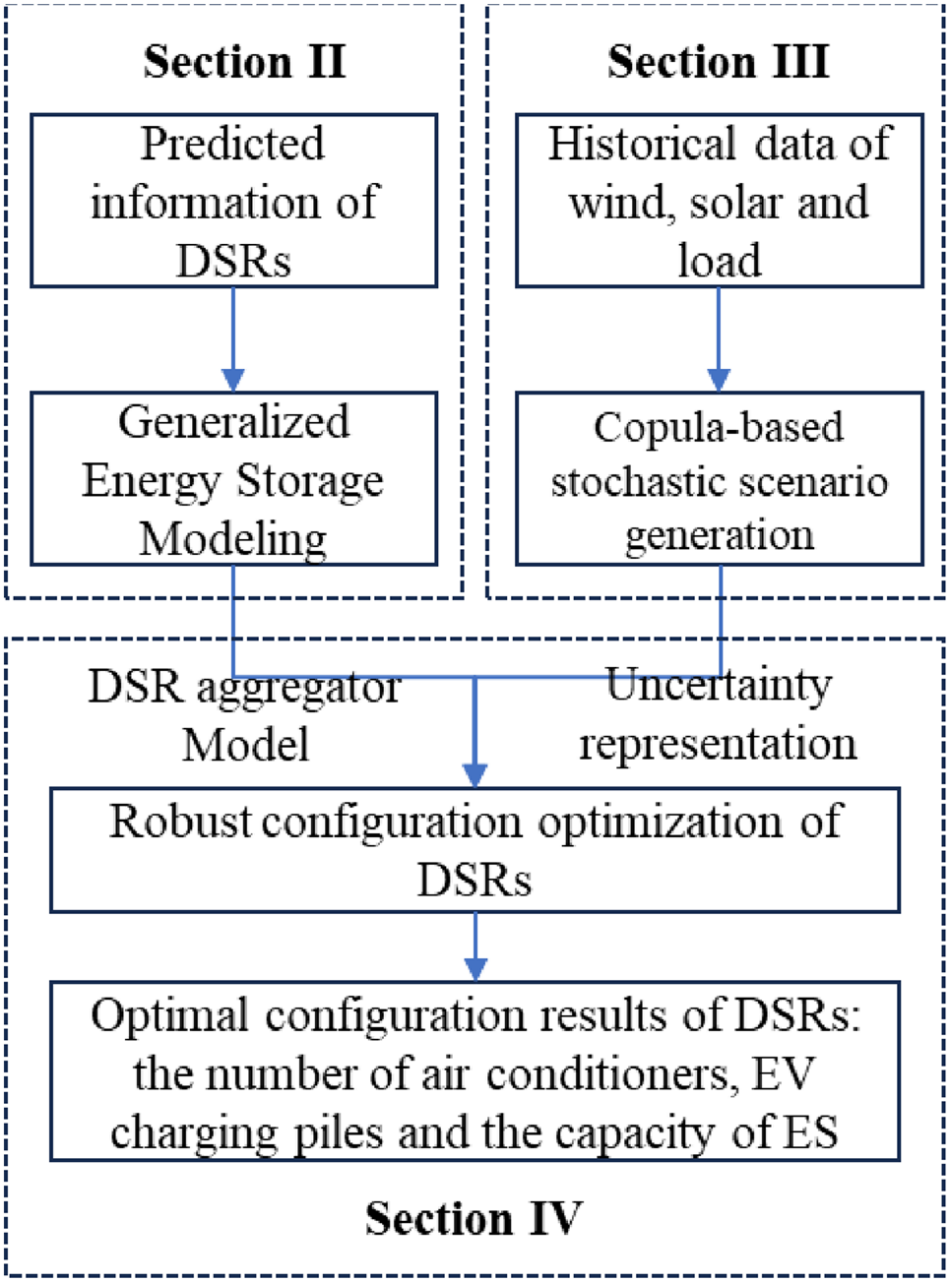
Over flow of the proposed method.

The overall flow of the algorithm is shown in Fig. 1. Generalized energy storage modeling for DSR aggregators and Copula-based stochastic scenario generation is conducted firstly. With the obtained DSR aggregator models and the uncertainty representations, the robust configuration optimization for DSRs is carried out. The step-by-step process is detailed as:

Step 1 The predicted information (outdoor temperature, users’ thermal comfort limits, charging demands of EVs) and device parameters (parameters of ACs, EV batteries and ESs) for DSRs are collected and utilized as inputs for GBM parameter extraction (Section "Aggregate flexibility modeling for demand-side resources");
Step 2 Based on the historical data of wind power, solar power and load consumption, Copula function is adopted to model the multivariable distribution with consideration of the dependence structure of wind power, solar power and load consumption. Stochastic scenarios sampling considering multivariable correlations is then achieved using Monte Carlo sampling from the predicted multivariable distribution (Section "Scenario generation considering wind, solar, and load correlations");
Step 3 After getting the DSR aggregator models based on GBM and the uncertainty representations based on scenario set, the robust optimization model is built as presented in Section "Robust optimal configuration method of demand-side flexible resources";
Step 4 Using commercial solution software to solve the optimization problem, the optimal configuration results are obtained, which is consist of the number of ACs, EV charging stations for retrofit, and additional ES capacity.

The above optimization models are linear programming models, which can be solved efficiently with the help of commercial solution software (e.g. Gurobi, Cplex).

## Example analysis

### Case setup

To verify the effectiveness of demand-side flexible resources in delaying grid transformation, reducing investment costs, and improving grid consumption, we applied the proposed method to optimize the configuration of demand-side flexible resources in Jiangsu province as an example. The installed capacities of wind, PV, and coal power planned for the province from 2025 to 2031 are shown in Table 1, and the investment/remodeling costs and maximum allocated capacities of each type of demand-side resources are presented in Table 2. According to Table 1, the installed capacity of coal-fired units shows a slight upward trend. The reason for the continuous increase in the installed capacity of coal-fired units is because, in the foreseeable future, coal-fired units remain irreplaceable. Despite the abundance of demand-side resources, the flexibility provided by individual resources is very limited, and they exhibit a high degree of randomness. It is unreliable and impractical to rely entirely on demand-side resources for achieving 100% renewable energy integration.

**Table 1:** 2025–2031 installed capacity of wind power, photovoltaic and coal power planning in Jiangsu Province.

Year	Wind power/MW	Solar/MW	Coal power/MW
2025	28,000	35,000	86,750
2026	30,000	46,000	88,750
2027	32,000	58,000	90,750
2028	35,400	65,000	92,750
2029	38,800	70,000	94,750
2030	45,000	75,000	96,750
2031	48,500	83,000	98,750

**Table 2:** Investment/modification costs and maximum allocation capacity for each type of demand-side resource.

Distributed energy resources	Investment/retrofit costs	Maximum capacity/size
Grid-side energy storage	3000RMB/kWh	30GWh
Electric Vehicles	3500RMB/pile	1.6 million
Air Conditioning	2500RMB/set	15 million units/25 million units

### Stochastic scenario generation results

**Figure 2:**
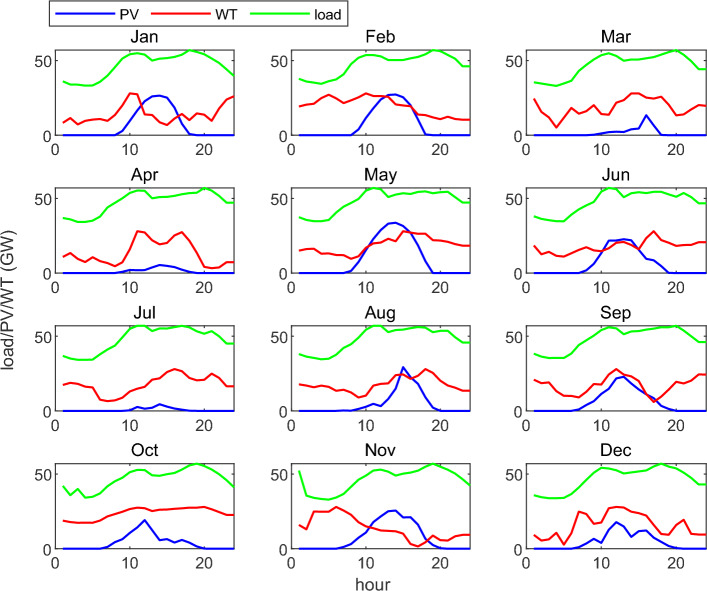
Typical daily fluctuation curves of wind, solar and load during the year.

Using a method for generating random scenarios that takes into account the correlation of random variables, we can derive N typical scenarios that depict the fluctuations in renewable energy and inflexible load. To streamline the optimization process, we've set the number of typical scenarios to 12, representing the 12 months in a year. The outcomes of the scenario generation are illustrated in Fig. 2.

Figure 2 displays the sampling results of wind, solar, and load random scenarios. These results reveal that among the three types of random factors, wind power output exhibits the strongest uncertainty due to various environmental factors like wind speed, temperature, and humidity. This uncertainty is prominently manifested in the significant fluctuations shown in the figure, displaying the most pronounced differences among different random scenarios. Influence by user electricity consumption behavior, the power curve randomness of the load comes next in intensity, presenting certain regularities. Specifically, it reaches peaks in electricity demand around 10:00 in the morning and approximately 20:00 in the evening due to user consumption patterns. In the case of photovoltaic power generation, influenced by factors such as sunlight intensity, temperature, and humidity, the power curve demonstrates relatively strong regularity. It consistently exhibits a characteristic shape, peaking around noon.

Overall, the random scenarios sampled based on the proposed methodology accurately and comprehensively cover the uncertainty distribution of supply and demand. These can effectively enhance the robustness and risk resistance of decision results in subsequent robust optimization.

### Demand-side flexible resource configuration results

**Figure 3:**
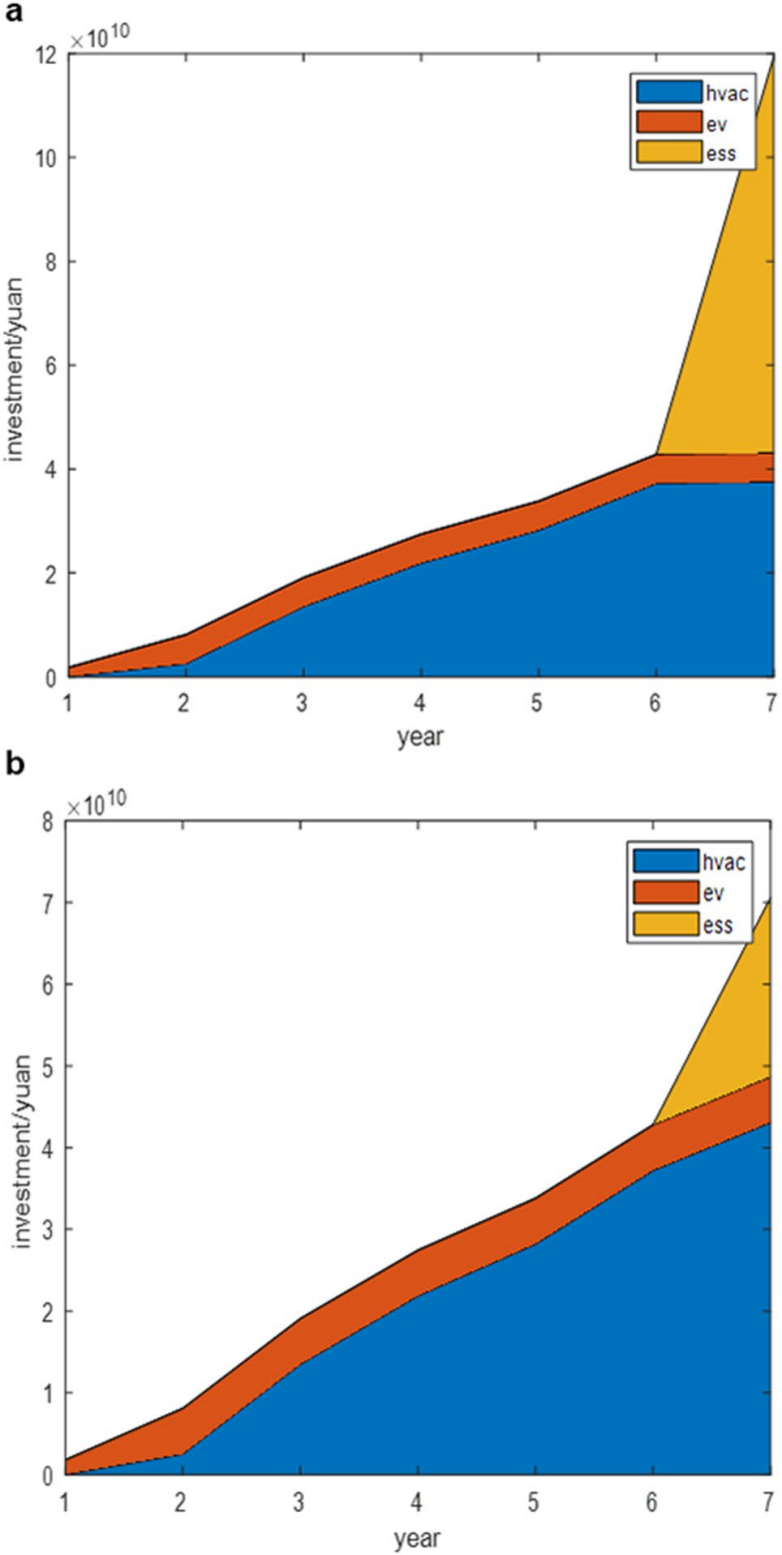
Cumulative investment cost curve 2025–2031. (**a**) 15 million units of air conditioners (**b**) 25 million units of air conditioners.

Based on the forecasted planned installed capacities of wind, PV, and coal power in Jiangsu Province from 2025 to 2031, we conducted year-by-year optimization for demand-side flexible resource allocation. The optimized configuration results are presented in Tables 3 and 4. To demonstrate the influence of various demand-side flexible resources on investment economics, different maximum retrofit table AC quantities are considered in Tables 3 and 4, respectively. Figure 3 depicts the cumulative investment cost curve and the cumulative investment cost of each component for the period 2025–2031.

**Table 3:** Annual planning for distributed energy resources 2025–2031 (15 million AC units).

Year	Wind power/MW	Solar/MW	Coal power/MW	Air conditioner/million units	Charging piles/million	Energy Storage/GWh	Total investment cost/billion yuan	Annual investment cost growth rate/billion yuan
2025	28,000	35,000	62,500	0.00	0.51	0.00	17.92	17.92
2026	30,000	46,000	64,000	1.00	1.60	0.00	80.98	63.06
2027	32,000	58,000	66,000	5.40	1.60	0.00	190.94	109.96
2028	35,400	65,000	68,000	8.74	1.60	0.00	274.58	83.64
2029	38,800	70,000	70,000	11.28	1.60	0.00	338.08	63.50
2030	45,000	75,000	72,000	14.88	1.60	0.00	428.05	89.97
2031	48,500	83,000	75,000	15.00	1.60	25.44	1194.32	766.27

**Table 4:** Annual planning for distributed energy resources 2025–2031 (25 million AC units).

Year	Wind Power/MW	Solar/MW	Coal power/MW	Air conditioner/million units	Charging iles/million	Energy Storage/GWh	Total investment cost/billion yuan	Annual investment cost growth rate/billion yuan
2025	28,000	35,000	62,500	0.00	0.51	0.00	17.92	17.92
2026	30,000	46,000	64,000	1.00	1.60	0.00	80.98	63.06
2027	32,000	58,000	66,000	5.40	1.60	0.00	190.94	109.96
2028	35,400	65,000	68,000	8.74	1.60	0.00	274.58	83.64
2029	38,800	70,000	70,000	11.28	1.60	0.00	338.08	63.50
2030	45,000	75,000	72,000	14.88	1.60	0.00	428.05	89.97
2031	48,500	83,000	75,000	17.23	1.60	7.31	706.13	278.08

As indicated in Table 3, the period from 2025 to 2030 demonstrates a steady and balanced increase in the number of air conditioners and electric vehicles in the absence of energy storage. However, as the scale of electric vehicle and air conditioner upgrades approaches its maximum limit in 2031, the grid is compelled to address the mounting pressure of renewable energy consumption by integrating energy storage. In 2031, a critical juncture is reached where the grid grapples with the necessity of incorporating energy storage due to the saturation of electric vehicle and air conditioner upgrades. However, it's important to note that this strategic shift comes at a cost. The investment required for energy storage significantly outweighs the transformation expenses associated with other distributed energy resources at this stage. Consequently, there is a remarkable upswing in the investment costs for power grid renovation in 2031, clearly depicted in Fig. 3a where the cumulative investment cost curve for the grid displays a sharp incline during this pivotal year. In summary, the flexibility and relatively modest retrofitting costs of air conditioning and electric vehicles play a pivotal role in extending the need for substantial grid retrofitting investments. This delay in major investments underscores the efficacy of leveraging the adaptability and cost-effectiveness of air conditioning and electric vehicles to manage the burgeoning demands on the power grid.

To further illustrate the significant role of demand-side flexible resources in postponing investments for grid upgrades, we increased the upper limit of air conditioner retrofitting scale from 15 to 25 million, and compared the investment costs between these two scenarios. A comparison between Tables 3 and 4 reveals that with the increase in the upper limit of air conditioner retrofitting scale, the cumulative investment cost by 2031 decreased from 1194.32 billion to 706.13 billion, reducing by approximately 42.2%. This clearly underscores the pivotal role of demand-side flexible resources in deferring investments for grid upgrades. It emphasizes the importance of fully exploiting the flexibility potential of demand-side resources and utilizing policies, markets, and other means to encourage active participation from demand-side users. This approach facilitates a win–win situation for the supply, demand, and grid stakeholders.

## Conclusion

In this paper, we propose an optimal configuration method for demand-side flexible resources to enhance renewable energy consumption. Firstly, we model the adjustable flexibility of demand-side resources based on the generalized battery model. Secondly, we generate random scenarios of wind, solar, and load with variable correlations using non-parametric probability prediction results of random variables combined with Copula function sampling. Next, we establish an optimal configuration model for demand-side flexible resources based on an improved robust optimization method. Finally, we optimize the demand-side resource renovation plan on an annual basis, considering the growth trend of installed renewable energy capacity in Jiangsu Province from 2025. The simulation results verify that the utilization of demand-side flexible resources can efficiently mitigate the costly investment in energy storage equipment.

Under price-based indirect control strategies, user responsiveness to prices is a significant factor influencing the adjustability of flexible resources. For our future work, users’ demand elastic to dynamic pricing strategy will be investigated by considering interactions between the aggregator and users.

## Data Availability

The datasets used and/or analyzed during the current study available from the corresponding author on reasonable request.

## References

[1] Lu Z, Li H, Qiao Y (2017). Flexibility evaluation and supply/demand balance principle of power system with high-penetration renewable electricity. Proc. CSEE.

[2] Ortega-Vazquez, M., Bouffard, F. & Silva, V. Electric vehicle aggregator/system operator coordination for charging scheduling and services procurement. In *2013 IEEE Power & Energy Society General Meeting*, July 21–25, Vancouver, Canada (2013).

[3] Lu N, Chassin DP, Widergren SE (2005). Modeling uncertainties in aggregated thermostatically controlled loads using a state queueing model. IEEE Trans. Power Syst..

[4] Chen X, Dall’anese E, Zhao CH (2020). Aggregate power flexibility in unbalanced distribution systems. IEEE Trans. Smart Grid.

[5] Hao H, Sanandaji BM, Poolla K (2015). Aggregate flexibility of thermostatically controlled loads. IEEE Trans. Power Syst..

[6] Zhao L, Zhang W, Hao H (2017). A geometric approach to aggregate flexibility modeling of thermostatically controlled loads. IEEE Trans. Power Syst..

[7] Yi ZK, Xu YL, Gu W (2021). Aggregate operation model for numerous small-capacity distributed energy resources considering uncertainty. IEEE Trans. Smart Grid.

[8] Li TX, Sun B, Chen Y (2021). Learning-based predictive control via real-time aggregate flexibility. IEEE Trans. Smart Grid.

[9] Wen YL, Hu ZC, You S (2022). Aggregate feasible region of DERs: Exact formulation and approximate models. IEEE Trans. Smart Grid.

[10] Tang X, Hu Y, Geng Q (2021). Multi-timescale optimal scheduling of integrated energy system considering multi-energy flexibility. Autom. Electr. Power Syst..

[11] Gong X (2020). Robust hierarchical control mechanism for aggregated thermostatically controlled loads. IEEE Trans. Smart Grid.

[12] Yi Z (2019). A multi-time-scale economic scheduling strategy for virtual power plant based on deferrable loads aggregation and disaggregation. IEEE Trans. Sustain. Energy.

[13] Xu Z (2016). Hierarchical coordination of heterogeneous flexible loads. IEEE Trans. Power Syst..

[14] Gao S, Liu S, Liu Y (2019). Flexible and economic dispatching of AC/DC distribution networks considering uncertainty of wind power. IEEE Access.

[15] Liu W, Li H, Zhang H (2018). Expansion planning of transmission grid based on coordination of flexible power supply and demand. Autom. Electr. Power Syst..

[16] Zhang G, Li F, Xie C (2020). Flexible robust risk-constrained unit commitment of power system incorporating large scale wind generation and energy storage. IEEE Access.

[17] Wang H, Wang S, Pan Z (2018). Optimized dispatching method for flexibility improvement of distribution network with high-penetration distributed generation. Autom. Electr. Power Syst..

[18] Chen R, Wang J, Botterud A (2017). Wind power providing flexible ramp product. IEEE Trans. Power Syst..

[19] Zhang M, Zhou M, Li G (2020). Quantifying accommodated domain of wind power for flexible look-ahead unit commitment. Electr. Power Syst. Res..

[20] Huo Y, Bouffard F, Joos G (2020). Spatio-temporal flexibility management in low-carbon power systems. IEEE Trans. Sustain. Energy.

[21] Lu Z, Li H, Qiao Y (2017). Flexibility evaluation and supply/demand balance principle of power system with high-penetration renewable electricity. Proc. CSEE.

[22] Wang Z, Shen C, Liu F (2018). An adjustable chance-constrained approach for flexible ramping capacity allocation. IEEE Trans. Sustain. Energy.

[23] Nyeng P, Jacob O (2011). Information and communications systems for control-by-price of distributed energy resources and flexible demand. IEEE Trans. Smart Grid.

[24] Hassan MAS (2019). Optimization modeling for dynamic price based demand response in microgrids. J. Clean. Prod..

[25] Yu X, Zhenyu D, Dandan Z (2023). Research on critical peak price decision optimization considering industrial consumer’s risk appetite under the carbon neutrality goal. Sustainability.

[26] Yu X, Dandan Z (2020). Cross-regional integrated energy system scheduling optimization model considering conditional value at risk. Int. J. Energy Res..

[27] Hamed S, Amir A, Mohammad SH (2020). Optimal demand response strategies to mitigate wind power variability and gas-supply uncertainty in a multi-resolution robust security constrained unit commitment. IET Gener. Transm. Distrib..

[28] Qu H, Zhu Y, Yin M (2017). Study on the unit commitment considering wind power paralleling in the power system. Power Syst. Prot. Control.

[29] Lu R, Ding T, Qin B (2020). Multi-stage stochastic programming to joint economic dispatch for energy and reserve with uncertain renewable energy. IEEE Trans. Sustain. Energy.

[30] Tian Y, Wu W, Wang KY (2020). Robust transmission constrained unit commitment under wind power uncertainty with adjustable conservatism. IET Gener. Transm. Distrib..

[31] Tumuluru VK, Tsang DHK (2018). A two-stage approach for network constrained unit commitment problem with demand response. IEEE Trans. Smart Grid.

[32] Zhang Z, Chen Y, Liu F (2021). Two-stage robust unit commitment model considering operation risk and demand response. Proc. CSEE.

[33] Jagdesh K, Chethan P, Mikko V (2020). Sizing and allocation of battery energy storage systems in Aland islands for large-scale integration of renewables and electric ferry charging stations. Energies.

[34] Yuan X, Fei J, Hu B (2019). Joint scheduling model of distributed generation, energy storage and flexible load under resource aggregator mod. Power Syst. Prot. Control.

[35] Wang ZY, Kirschen DS (2019). Two-stage optimal scheduling for aggregators of batteries owned by commercial consumers. IET Gener. Transm. Distrib..

[36] Frazier AW, Cole W, Denholm P (2020). Assessing the potential of battery storage as a peaking capacity resource in the United States. Appl. Energy.

[37] Denholm P, Nunemaker J, Gagnon P (2020). The potential for battery energy storage to provide peaking capacity in the United States. Renew. Energy.

[38] Cui Y, Zhou H, Zhong W (2021). Optimal dispatch of power system with energy storage considering deep peak regulation initiative of thermal power and demand response. High Volt. Eng..

[39] Wang S, Chen J, Wang H (2020). Two-stage flexibility improvement optimization method of distribution network considering EV charging and scheduling of energy storage and interruptible loads. Electr. Power Autom. Equip..

[40] Ma W, Gao H, Li H (2019). Flexibility evaluation and optimal dispatch model of distribution network considering soft open point. Power Syst. Technol..

[41] Wu J, Ai X, Hu J (2019). Optimal dispatch of flexible resource on demand side considering uncertainties. Autom. Electr. Power Syst..

[42] Li Z, Li T, Wu W (2019). Minkowski sum based flexibility aggregating method of load dispatching for heat pumps. Autom. Electr. Power Syst..

